# Pharmacokinetics of the Antiviral Lectin Griffithsin Administered by Different Routes Indicates Multiple Potential Uses

**DOI:** 10.3390/v8120331

**Published:** 2016-12-17

**Authors:** Christopher Barton, J. Calvin Kouokam, Harrell Hurst, Kenneth E. Palmer

**Affiliations:** 1 Department of Pharmacology and Toxicology, University of Louisville School of Medicine, Louisville, KY 40202, USA; christopher.barton@louisville.edu (C.B.); j0kouo01@louisville.edu (J.C.K.); hehurs01@louisville.edu (H.H.); 2 James Graham Brown Cancer Center, University of Louisville School of Medicine, Louisville, KY 40202, USA; 3 Center for Predictive Medicine, University of Louisville School of Medicine, Louisville, KY 40202, USA

**Keywords:** Griffithsin, pharmacokinetics, *per os*, systemic administration, rat model

## Abstract

Griffithsin (GRFT) is a red alga-derived lectin with demonstrated broad spectrum antiviral activity against enveloped viruses, including severe acute respiratory syndrome–Coronavirus (SARS-CoV), Japanese encephalitis virus (JEV), hepatitis C virus (HCV), and herpes simplex virus-2 (HSV-2). However, its pharmacokinetic profile remains largely undefined. Here, Sprague Dawley rats were administered a single dose of GRFT at 10 or 20 mg/kg by intravenous, oral, and subcutaneous routes, respectively, and serum GRFT levels were measured at select time points. In addition, the potential for systemic accumulation after oral dosing was assessed in rats after 10 daily treatments with GRFT (20 or 40 mg/kg). We found that parenterally-administered GRFT in rats displayed a complex elimination profile, which varied according to administration routes. However, GRFT was not orally bioavailable, even after chronic treatment. Nonetheless, active GRFT capable of neutralizing HIV-Env pseudoviruses was detected in rat fecal extracts after chronic oral dosing. These findings support further evaluation of GRFT for pre-exposure prophylaxis against emerging epidemics for which specific therapeutics are not available, including systemic and enteric infections caused by susceptible enveloped viruses. In addition, GRFT should be considered for antiviral therapy and the prevention of rectal transmission of HIV-1 and other susceptible viruses.

## 1. Introduction

Several lectins isolated from natural sources inhibit microbial and viral pathogens both in vitro and in vivo [1,2,3,4,5,6]. Griffithsin (GRFT), derived from the red alga *Griffithsia* sp., is a homodimeric lectin with a total of six oligosaccharide binding sites recognizing terminal oligomannose residues on asparagine (N)-linked Man_5-9_-GlcNAc_2_ structures [7,8,9,10,11]. Interestingly, oligomannose structures constitute the vast majority of N-linked glycans in the HIV-1 glycan shield [12,13], and help the virus evade immune surveillance [14]. Griffithsin inhibits HIV-1 in vitro with an EC_50_ in the mid-picomolar range [7,15,16], with nanomolar activity against several other enveloped viruses such as acute respiratory syndrome-coronavirus (SARS-CoV), Japanese encephalitis virus (JEV), hepatitis C virus (HCV), and herpes simplex virus-2 (HSV-2) [3,17,18,19]. GRFT binds oligosaccharides to multiple sites on a single molecule [11], thereby occluding functionally important domains of the viral envelope glycoprotein, preventing interactions with cell-surface receptors and impeding structural transitions indispensable for infection initiation. As demonstrated with HSV-2, GRFT may also act by inhibiting virus egress and spread post-infection in vitro [17]. Importantly, GRFT’s antiviral activity has been confirmed in pre-exposure prophylaxis animal models, with the lectin inhibiting HSV-2, SARS-CoV, JEV, and HCV [3,17,18,19,20,21]. This potent activity of GRFT is coupled with an excellent safety profile as shown in vitro and in several animal models [15,22,23]. Taken together, GRFT’s tolerability profile and broad spectrum activity against susceptible viruses, both in vitro and in vivo, advocate for its further development as an antiviral chemopreventative or chemotherapeutic.

Despite the impressive wealth of studies assessing GRFT’s therapeutic value, little is known about its pharmacokinetic profile, which could limit its use as a drug. Therefore, this study aimed to evaluate the pharmacokinetic properties of this promising antiviral, after administration by different routes.

We found that GRFT was readily bioavailable in rats after intravenous and subcutaneous treatments, showing distinct pharmacokinetic profiles according to the route employed. However, GRFT was not detected in serum after oral single or chronic dosing; nevertheless, a small fraction of orally-administered GRFT formulated in PBS was found in fecal extracts in its active form. Our findings indicate that GRFT’s pharmacokinetics depends on the administration route, while indicating a potential multi-use of this lectin for the treatment of systemic and enteric infections caused by susceptible enveloped viruses.

## 2. Materials and Methods

### 2.1. Lectin Reagents

Recombinant GRFT produced in *Nicotiana benthamiana* plants as described previously [15] was purified to >99% purity in phosphate-buffered saline (PBS, pH 7.4) with <0.05 endotoxin units (EU) per milligram.

### 2.2. Animal Housing and Care

Sprague Dawley (SD) rats (*Rattus norvegicus*, Charles River Laboratories, USA) weighing 250 g were housed in a temperature- and humidity-controlled room with an alternating light/dark cycle of 12 h, with standard diet and water *ad libitum*. All animal procedures were approved by the University of Louisville’s Institutional Animal Care and Use Committee.

### 2.3. Pharmacokinetic Study and Sample Collection after Single Dose Administration

SD rats (250 g) were procured from Charles River with indwelling femoral vein catheters. After a week of adaptation, the animals were weighed and treated with a single dose of GRFT by different routes. For intravenous treatment, indwelling catheters were checked for patency and either 10 or 20 mg/kg GRFT was infused through the catheter over a 15 s period. For subcutaneous treatment, 10 or 20 mg/kg GRFT was injected under the skin between the shoulders. For oral dosing, 10 mg/kg of GRFT formulated in PBS at a final volume of 1 mL was administered by gavage.

Approximately 150 µL of blood were collected from each intravenously and subcutaneously dosed animal via the indwelling femoral catheter at the following time points after GRFT administration: 15 min, 30 min, 1 h, 2 h, 4 h, 8 h, 24 h, 48 h, 72 h, and 96 h. In the case of oral treatment, 150 µL of blood were drawn 10 min, 20 min, 30 min, 1 h, 2 h, 4 h, 6 h, 24 h, and 24 h post-administration. All animals were sacrificed by CO_2_ asphyxiation followed by thoracotomy at study completion.

### 2.4. Active Mass Balance Rat Treatment and Sample Collection

Rats were treated with a single dose of 10 mg/mL GRFT either intravenously (n = 5) through the indwelling catheter or subcutaneously (n = 5). Additional rats were treated with 10 mg/mL GRFT in 1 mL solution by gavage. Post-dosage, animals were placed in metabolic cages and urine samples were collected at 4, 8, 12, 20, and 48 h; fecal pellets were collected at 24 and 48 h. Urine volumes and fecal pellet masses were recorded for mass balance calculations.

### 2.5. Chronic Oral Dosing Treatment and Sample Collection

A total of 18 rats with no indwelling catheters were treated daily with PBS (n = 6), 20 mg/kg GRFT (n = 6), or 40 mg/kg GRFT (n = 6) for a period of 10 days by gavage of 1 mL solution. Blood samples were drawn via lateral tail vein on day 5 approximately 3 h after oral dosing. On day 10, animals were sacrificed approximately 3 h after treatment by CO_2_ asphyxiation, followed by thoracotomy; blood samples were then obtained by cardiac puncture. Urine samples were collected, as well, at selected time points after oral dosing.

Six fecal pellets were randomly collected at 8 and 24 h after initial treatment, and at selected days thereafter. Briefly, 2 mL PBS were added per gram of pellets 15–30 min before mixing. The homogenates were cleared twice by centrifugation at 4 °C for 10 min at 1000× *g*. The resulting fecal suspension was stored at −20 °C, for the determination of GRFT concentrations and anti-HIV activity. Fecal extract samples were pooled by combining equal volumes of extracts from each cage within the applicable treatment group. Fecal extracts from chronically treated animals underwent additional processing to further remove contaminants before HIV-1 pseudovirus neutralization assays: samples were sterile filtered using Costar Spin-X centrifuge tube filters containing 0.22 µm cellulose acetate filters. After filtration, samples were dialyzed at 2 kDa molecular weight cut off against PBS for 24 h at 4 °C.

Extracts for mass balance experiments were prepared by desiccation and rehydration of fecal pellets. After overnight desiccation, pellets were pulverized using a commercial coffee grinder. Pellet extracts were formulated using 0.5 g of dried powder and 2 mL of PBS. The resulting fecal slurry was thoroughly mixed and cleared twice by centrifugation at 1000× *g* for 5 min; the final supernatant was aliquoted for GRFT concentration determination.

### 2.6. GRFT Capture Immunoassay Using Purified Influenza Hemagglutinin

To detect trace amounts of GRFT in serum and fecal extracts an enzyme-linked immunosorbent assay (ELISA) was carried out as previously described [22], coating plates with 10 µg/mL purified influenza hemagglutinin (HA, Kentucky Bioprocessing, Owensboro, KY, USA) instead of 250 ng/mL gp120. This method detects active GRFT.

### 2.7. Evaluation of Anti-HIV Activity of Fecal Extracts

HIV-1 neutralization activity was assessed of pooled fecal extracts from chronically treated animals as previously described [24]. Briefly, DU156 Env-pseudotyped virus particles were obtained by transfection of 293T cells and titrated in TZM-bl cells. Antiviral effect was reflected by luciferase reporter gene activity, determined by relative luminescence after substrate addition. The 50% inhibitory dose (ID50) represented the sample dilution required to reduce luminescence by half in comparison to control wells.

### 2.8. Histopathology

Organs, including liver, kidney, heart, lung, and spleen, were obtained from each animal and paraffin embedded after fixation in 10% neutral buffered formalin. Then, sections were stained with hematoxylin and eosin (H and E) and evaluated in a blinded manner by a veterinary pathologist (Dr. Mary Proctor, University of Louisville) for gross or cellular abnormalities.

### 2.9. Pharmacokinetic Analysis

Pharmacokinetic parameters were obtained with PK Solutions Software (SummitPK, Eugene, OR, USA), using GRFT serum concentrations at sampling time points. Values were generated from concentration time curves based upon GRFT amounts administered, and included absorption, distribution, elimination half-life, area under the curve (AUC), volume of distribution (Vd), clearance, and maximum serum concentration (C_max_).

### 2.10. Statistical Analyses

Graph Pad Prism 5 and SAS software version 9.3 were used for statistical analyses. *P* < 0.05 was considered statistically significant.

## 3. Results

### 3.1. GRFT’s Bioavailability after Intravenous Administration

**Figure 1:**
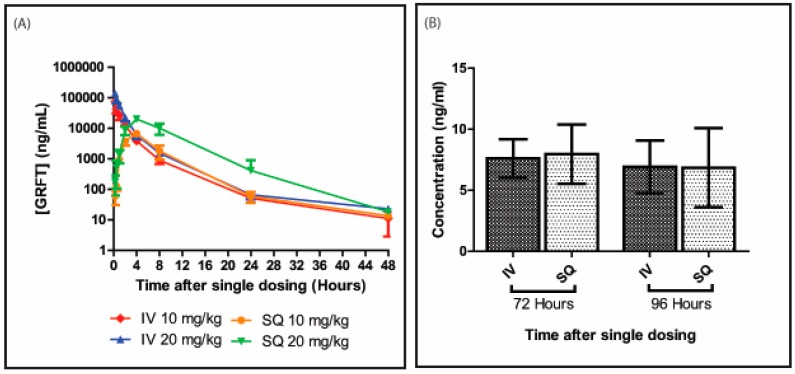
Pharmacokinetic profile of GRFT. Mean serum GRFT concentrations after single intravenous (IV) or subcutaneous (SQ) administration (n = 3–4 per group) (**A**). Serum GRFT amounts in animals dosed with 10 mg/kg were assessed at both 72 and 96 h post-administration (**B**). Bars are mean ± standard deviation from five biological replicates.

GRFT was detected at all time points assessed after administration of a single dose (Figure 1). As shown in Figure 1A, serum concentrations increased with lectin dose. At 15 min, animals displayed average serum GRFT concentrations of 74 ± 8 and 141 ± 22 µg/mL in the 10 and 20 mg/kg groups, respectively. These amounts rapidly decreased over time, with 827 ± 256 vs. 1540 ± 312 ng/mL, 54 ± 12 vs. 66 ± 19 ng/mL, and 10 ± 7 vs. 23 ± 4 ng/mL at 8, 24, and 48 h post-administration, respectively. Interestingly, GRFT remained detectable in serum at low concentrations up to 96 h post-treatment (Figure 1B).

### 3.2. GRFT’s Bioavailability after Subcutaneous Administration

After subcutaneous administration, serum GRFT amounts gradually increased from 61 ± 30 and 171 ± 110 ng/mL at 15 min for the 10 and 20 mg/kg groups, respectively, to peak at approximately 4 h post-administration with 6615 ± 602 and 19740 ± 2376 ng/mL, respectively (Figure 1A). This was followed by a decreasing trend, similar to that observed in intravenously dosed animals. At 8, 24, and 48 h, 1811 ± 883, 59 ± 23, and 14 ± 2 ng/mL GRFT, respectively, were detected in the low dose treatment group, while 10114 ± 3988, 411 ± 479, and 18 ± 3 ng/mL were obtained after drug administration at 20 mg/kg, respectively. GRFT was detected in serum samples from both treatment groups at 96 h post-treatment (Figure 1B).

### 3.3. GRFT’s Pharmacokinetic Parameters after Parenteral Administration

Table 1 summarizes the various pharmacokinetic parameters of GRFT over 48 h. Analysis of concentration-time curves with the PK Solutions software after intravenous administration revealed a multiphasic kinetics, with three distinct phases: absorption, distribution, and elimination. Half-lives for absorption (0–2 h), distribution (4–8 h) and elimination (24 h and up) were 0.5 ± 0.1 h, 1.7 ± 0.3 h, and 10.7 ± 4.6 h, respectively, in animals treated intravenously with 10 mg/kg GRFT; similar values were obtained after high dose administration by the same route.

After subcutaneous administration of 10 mg/kg GRFT, these pharmacokinetic parameters showed an increasing trend: 1.3 ± 0.3 h, 2.1 ± 0.9 h, and 13.8 ± 6.8 h were obtained as absorption, distribution, and elimination half-lives, respectively; similar values were obtained with the high dose of 20 mg/kg for these parameters. AUC values were higher in intravenously administered animals than in the subcutaneous group for both low (105.7 ± 16.9 mg×h/L vs. 45.6 ± 9.9 mg×h/L) and high (203.6 ± 27.6 mg×h/L vs. 183.2 ± 45.3 mg×h/L) GRFT doses. A similar trend was observed for maximum serum concentrations (C_max_). Intravenous administration of 10 and 20 mg/kg GRFT resulted in C_max_ values of 81.8 ± 25.7 and 176.0 ± 26.7 µg/mL, respectively; in subcutaneously administered animals, these values dropped to 6.6 ± 0.6 and 19.7 ± 2.4 µg/mL, respectively. Volumes of distribution (Vd) and clearance rates were relatively low for both routes and all doses tested.

### 3.4. Pharmacokinetic Parameters after Oral Administration

GRFT was not detected in serum at any time point after oral administration of a 20 mg/kg single dose. 

### 3.5. GRFT Is Found in Fecal Extracts after Chronic Oral Treatment

**Figure 2:**
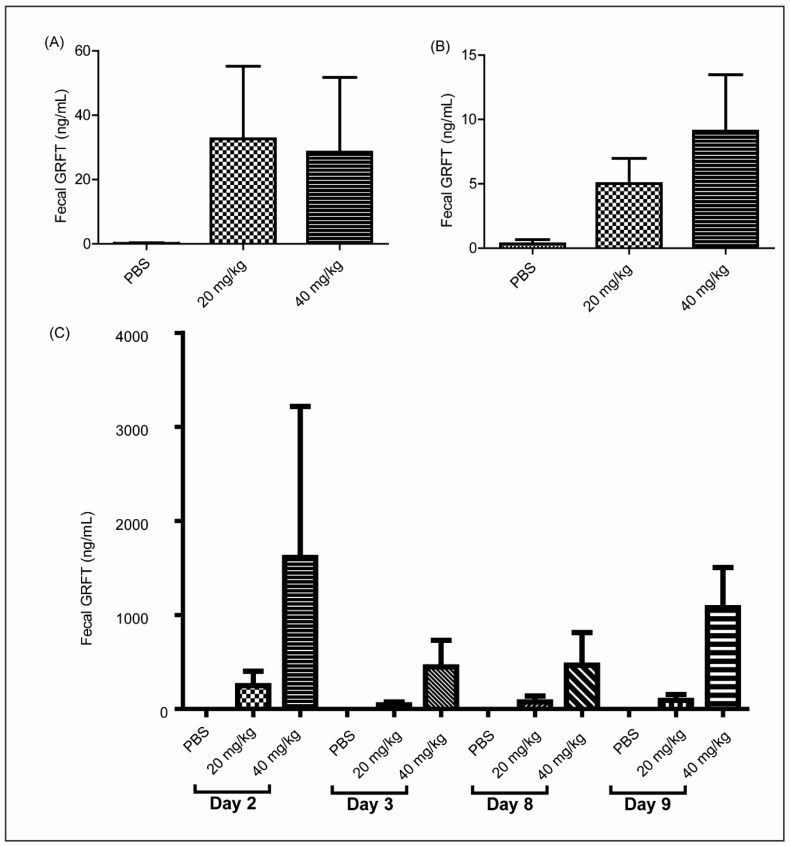
Fecal GRFT concentrations following chronic oral dosing. Fecal extracts were prepared from fresh fecal pellets obtained 8 h (n = 5) (**A**) and 24 h (n = 6) (**B**) after oral treatment with PBS, 20 mg/kg GRFT, or 40 mg/kg GRFT. Bars are mean ± standard deviation from five biological replicates. Fecal extracts (n = 3) were prepared from desiccated pellets randomly drawn from cages (two animals per cage) on indicated days (**C**); bars are mean ± standard deviation from three biological replicates.

At 8 h after initial dosing, the animals treated with 20 and 40 mg/kg GRFT displayed fecal GRFT concentrations averaging approximately 30 ng/mL (Figure 2A). However, these levels decreased in both groups at 24 h after dosing, with 5 and 9 ng/mL obtained in average in the 20 and 40 mg/kg GRFT treatment groups, respectively (Figure 2B). Fecal pellets were collected at days 2, 3, 8, and 9 during the 10 day treatment. Fecal extracts from animals treated with 20 mg/kg GRFT showed concentrations ranging from 44 to 250 ng/mL, and higher amounts were obtained after treatment with 40 mg/kg (447 to 1612 ng/mL) (Figure 2C).

### 3.6. Fecal Extracts after Chronic Oral Treatment Show Neutralization Activity Against HIV

**Figure 3:**
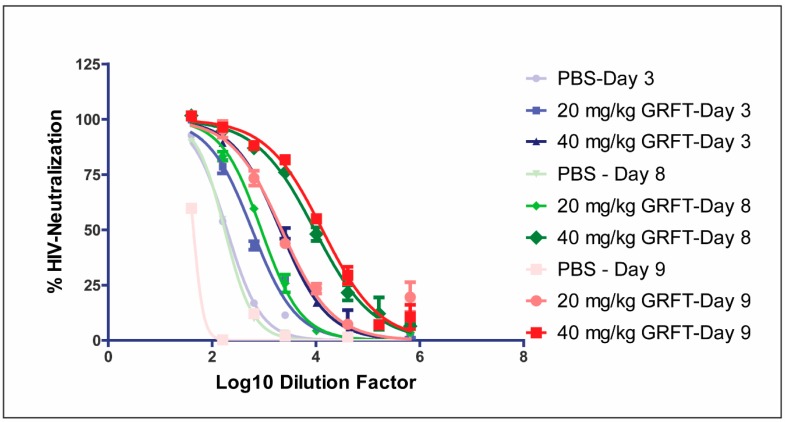
Antiviral activity of pooled fecal extracts collected during chronic oral treatment with GRFT. HIV-1 env-pseudovirus neutralization activity was assessed in pooled rat fecal extracts from animals dosed with 20 or 40 mg/kg GRFT, and expressed as ID_50_ (dilution factor required to reduce luminescence to 50% of PBS treated controls).

Fecal extracts from rats orally treated with 20 and 40 mg/kg GRFT neutralized HIV-1 pseudoviruses (Clade C primary sexually transmitted isolate Du156) with ID_50_ (dilution factor required to reduce luminescence to 50% of PBS treated controls) values of 590 and 2059, respectively, on day 3 (Figure 3 and Table 2). Interestingly, higher antiviral activity was obtained for 20 and 40 mg/kg doses at day 8 (ID_50_ 885 and 9452, respectively) and day 9 (ID_50_ 2195 and 13,307, respectively) of treatment (Table 2 and Figure 3).

### 3.7. Mass Balance of Active GRFT

**Figure 4:**
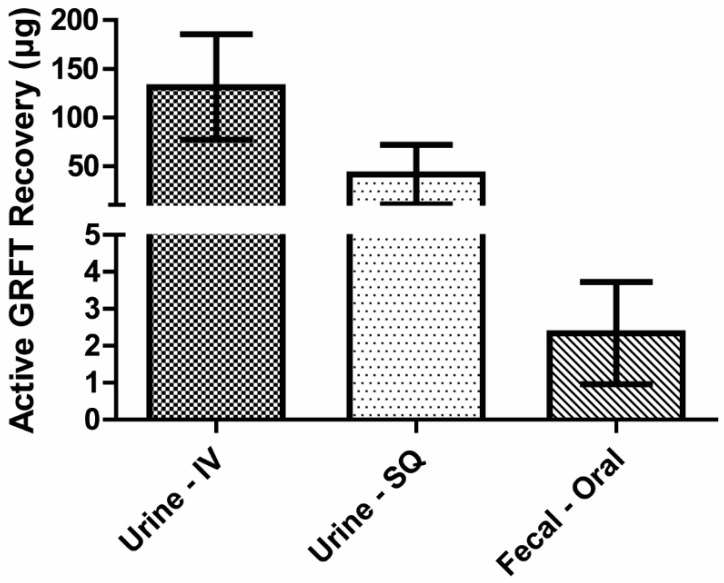
Total GRFT recovery following single administration. Based upon the likely route of excretion, urine or fecal samples were obtained from animals treated with 10 mg/kg GRFT intravenously (IV), subcutaneously (SQ), and orally. GRFT amounts in respective samples were determined. Bars indicate mean ± standard deviation from five biological replicates.

Concentrations of active GRFT were assessed in urine and fecal materials produced within 24 h following single intravenous, subcutaneous and oral treatments, respectively, of a 2.5 mg (approximately 10 mg/kg) dose. Intravenous and subcutaneous administrations resulted in 131 and 41 µg GRFT in urine, respectively (Figure 4). Meanwhile, urine GRFT levels in animals treated orally were below detection. Instead, GRFT was found in fecal extracts, and approximately 2.3 µg GRFT was recovered in the feces.

## 4. Discussion

The data described here confirmed our previous findings that subcutaneous GRFT administration is well tolerated in laboratory rodent species (mouse and guinea pig), accumulating to therapeutically relevant levels [22]. In addition, this work revealed a complex PK profile for GRFT in rats, a standard rodent model used for PK studies.

As shown above, GRFT’s drug concentration time curves varied with the administration route (Figure 1). Indeed, a multiphasic elimination pattern was observed after intravenous treatment, with GRFT’s serum half-life changing over time. Although most of the bolus GRFT was eliminated within 8 h, therapeutically effective amounts remained for at least 96 h post-administration. Such complex elimination patterns have been observed in other recombinant protein based pharmaceuticals, which show differences in time to maximum serum concentrations and elimination rates [25,26,27]. GRFT was excreted mainly in urine starting shortly after intravenous dosing, and urine concentrations decreased with time. Further *in vivo* radiolabeling studies are required to determine the full GRFT distribution in the body. In addition, degradative proteolysis of the lectin might be occurring. Previous studies have shown GRFT is relatively resistant to most proteases; however, it is susceptible to leukocyte elastase [28]. Therefore, GRFT might also be vulnerable to yet to be tested proteases: extensive in vitro protease degradation assays and catabolite assessment in serum after systemic administration are needed to fully understand GRFT’s susceptibility to proteases.

As shown above, the final phase of GRFT’s elimination half-life is comparable to that of other protein therapeutics such as monoclonal antibodies [26] and smaller molecule biologics [25]. Serum persistence could potentially result from redistribution from deeper physiological compartments. However, it is more likely that GRFT is involved in interactions with endogenous proteins. These hypotheses should be tested in vivo with radiolabeled GRFT and cellular protein binding experiments. Subcutaneous GRFT treatment resulted in serum concentrations peaking at approximately 4 h post-administration, with the elimination patterns mirroring intravenous treatment thereafter (Figure 1A). The initial distribution phase immediately following subcutaneous dosage was fairly comparable for both GRFT doses (10 and 20 mg/kg). However, the high dose resulted in markedly increased GRFT’s peak concentration, total drug exposure, and bioavailability, compared with the low dose, as reflected by AUC values. The approximately four-fold increase in total drug amounts obtained between 10 and 20 mg/kg doses could be attributable to a gradual release or absorption from the injection site over time, while competing distribution, elimination, and degradative processes occur as the lectin enters the circulation from the capillary bed.

Since serum GRFT concentrations peaked at 4 h post bolus dosing, and persisted up to 96 h, daily subcutaneous self-administration would maintain potential therapeutic effects. Understanding GRFT’s pharmacokinetics is very critical for future studies. Given that subcutaneous dosing may be viable in maintaining potentially therapeutic drug concentrations, the following parameters should be assessed: steady-state drug concentrations, loading doses, maintenance doses, and dosing intervals. GRFT’s EC_50_ for HIV is approximately 40 pM [7]. Based on 0.06 L/h clearance determined for single 10 mg/kg subcutaneous dose, administration of 2.5 mg/kg GRFT every 72 h would achieve a steady-state concentration of 47 nM, over 1000 times the EC_50_ for HIV-1. However, GRFT has higher EC_50_ for HCV (4 nM) [3] and SARS-CoV EC_50_ (48 nM) [19], indicating that optimal regimens should be determined for various viral diseases, maintaining serum drug concentrations at therapeutic steady-state levels while avoiding toxicities. More frequent regimens could also be designed based on these data, to minimize peak and trough drug levels while maintaining potentially effective steady-state serum concentrations. For instance, a steady state concentration of 47 nM would require 35 µg/h or 840 µg/day to be administered. Interestingly, GRFT amounts dosed subcutaneously affected its bioavailability: at 10 and 20 mg/kg, respectively, GRFT displayed 43 and 90% bioavailability rates (AUC_subcutaneous_)/AUC_intravenous_). Although the underlying reasons remain unclear, these findings highlight the impact of dose selection on study design. It should be noted that only active GRFT was detected in this study. Preliminary experiments in our laboratory have shown that GRFT may bind some proteins in the plasma. However, how such binding affects the actual detection of GRFT deserves further attention. Using more sensitive measurements of the lectin, e.g., mass spectrometry or performing treatments with the radiolabeled compound would provide additional insights. Such experiments will be carried out in future studies.

In contrast to parenteral routes, oral administration of GRFT did not yield detectable blood levels. Nevertheless, active GRFT concentrations present in feces indicate it could be used orally to prevent the rectal transmission of HIV-1 and other pathogens such as HSV-2. Indeed, the non-absorption of GRFT after oral administration allowed for a drug fraction to pass through the digestive tract, ultimately being expelled in fecal material (Figure 4). Approximately 1% of initial GRFT amount was recovered in the feces; shielding the lectin from stomach acid and protease effects might increase the excreted amounts. Nonetheless, GRFT fecal levels exceeded its EC_50_ toward HIV-1 within the first 24 h post treatment.

Daily prophylactic treatment for HIV-1 is well established, e.g., Truvada is currently prescribed to high risk populations, including men who have sex with men (MSM) [29,30]. Additionally, antiretrovirals are employed following occupational and non-occupational exposure to HIV [31,32,33,34,35]. Therefore, oral GRFT could be utilized daily to “seed” the bowels and rectal mucosa, potentially blocking HIV infection. Interestingly, GRFT passage following oral dosage was consistent with the typical intestinal transit time of 20–30 h [36]. Finally, given that serum GRFT concentrations peaked at 4 h post bolus parenteral dosing, and persisted up to 96 h, daily subcutaneous self-administration would maintain a potential therapeutic effect as a prophylactic for HIV.

## 5. Conclusions

The continuous worldwide spread of enveloped viruses, such as SARS-CoV, HIV-1, HSV-2, and HCV, demands robust prevention measures. GRFT’s pharmacokinetic profile supports multiple potential uses and enables the design of different regimens. As a systemic post-exposure treatment, subcutaneous self-administration would be both viable and effective, achieving potentially therapeutic serum concentrations of GRFT. In the context of post-exposure prophylaxis to emerging viral threats, an initial intravenous bolus treatment with GRFT may be useful to achieve speedy serum peak levels. In addition, GRFT could be utilized orally to prevent colorectal transmission of HIV and susceptible viruses in a pre-exposure prophylactic setting.

## Figures and Tables

**Table 1:** Pharmacokinetic parameters of Griffithsin (GRFT) within 48 h of systemic administration.

		Intravenous		Subcutaneous	
Parameter	Unit	10 mg/kg (n = 4)	20 mg/kg (n = 3)	10 mg/kg (n = 4)	20 mg/kg (n = 4)
absorption half life	hour	0.5 ± 0.1	0.5 ± 0.2	1.3 ± 0.3	1.6 ± 0.4
distribution half life	hour	1.7 ± 0.3	2.1 ± 0.7	2.1 ± 0.9	2.8 ± 1.2
elimination half life	hour	10.7 ± 4.6	17.5 ± 6.1	13.8 ± 6.8	6.6 ± 1.9
AUC	mg-h/L	105.7 ± 16.9	203.6 ± 27.6	45.6 ± 9.9	183.2 ± 45.3
VD	L	0.4 ± 0.1	0.6 ± 0.2	1.2 ± 0.6	0.2 ± 0.1
Clearance	L/h	0.03 ± 0.01	0.02 ± 0.01	0.06 ± 0.01	0.02 ± 0.01
C_MAX_	µg/mL	81.8 ± 25.7	176.0 ± 26.7	6.6 ± 0.6	19.7 ± 2.4

Data are mean ± standard deviation. Absorption, distribution, elimination half-life, area under the curve (AUC), volume of distribution (VD), clearance, and maximum serum concentration (Cmax) were determined.

**Table 2:** Antiviral activity of pooled fecal extracts collected during chronic oral treatment with GRFT.

Day	Treatment	Fecal GRFT (ng/mL)	Interpolated ID50
3	PBS	0	192
	20 mg/kg GRFT	23	590
	40 mg/kg GRFT	447	2059
8	PBS	0	174
	20 mg/kg GRFT	46	885
	40 mg/kg GRFT	460	9452
9	PBS	0	43
	20 mg/kg GRFT	140	2195
	40 mg/kg GRFT	1073	13,307

HIV-1 Env-pseudovirus neutralization activity was assessed in pooled rat fecal extracts from animals dosed with 20 or 40 mg/kg GRFT, and expressed as ID_50_ (dilution factor required to reduce luminescence to 50% of PBS-treated controls). The actual mean GRFT concentrations were determined in fecal extracts to derive experimental EC_50_ obtained from interpolated ID_50_ values.
